# Da Vinci Single-Port Transvaginal Natural Orifice Transluminal Endoscopic Surgery (vNOTES) Sacrocolpopexy: A Case Report and Literature Review

**DOI:** 10.7759/cureus.106596

**Published:** 2026-04-07

**Authors:** Xiaoming Guan, Chunhua Zhang, Qiannan Yang, Shunjia Hong, Wai Ieng Fong

**Affiliations:** 1 Obstetrics and Gynecology, Baylor College of Medicine, Houston, USA; 2 Obstetrics and Gynecology, The Second Affiliated Hospital of Nanjing Medical University, Nanjing, CHN; 3 Obstetrics and Gynecology, Conde S. Januário Hospital, Macau, CHN

**Keywords:** da vinci single-port system, pelvic organ prolapse, robotic-assisted surgery, sacrocolpopexy, vnotes

## Abstract

Pelvic organ prolapse remains a common condition requiring durable surgical correction, with sacrocolpopexy considered the gold standard. Transvaginal natural orifice transluminal endoscopic surgery (vNOTES) has emerged as a scarless minimally invasive approach, while the integration of robotic single-port technology may further enhance surgical precision and ergonomics. However, data on robotic-assisted single-port vNOTES (RSP-vNOTES) sacrocolpopexy remains limited. We report a case of a 58-year-old Asian woman with symptomatic apical pelvic organ prolapse. The pelvic organ prolapse quantification (POP-Q) assessment demonstrated stage II uterine prolapse. She underwent robotic-assisted single-port vNOTES (RSP-vNOTES) sacrocolpopexy using the da Vinci single-port (SP) platform. The procedure was successfully completed via a transvaginal approach without abdominal incisions. Operative time was 170 min, with minimal blood loss (25 mL), and no intraoperative complications occurred, nor was a conversion required. The patient was discharged on postoperative day two and demonstrated an uneventful recovery with sustained anatomical support at one-year follow-up. This case highlights the feasibility and safety of RSP-vNOTES sacrocolpopexy and underscores its potential advantages, including enhanced visualization, improved instrument dexterity, and the benefits of a scarless approach. As an early application of RSP-vNOTES in pelvic reconstructive surgery, this technique may represent a significant advancement in minimally invasive management of pelvic organ prolapse.

Therefore, this case report aimed to describe the surgical technique and perioperative outcomes of RSP-vNOTES sacrocolpopexy using the da Vinci SP platform, while highlighting its potential advantages and clinical relevance. By presenting this early experience, we seek to contribute to the evolving evidence base and inform future adoption of this innovative approach in minimally invasive pelvic reconstructive surgery.

## Introduction

Pelvic organ prolapse (POP) is a common condition characterized by the descent of pelvic structures, including the anterior or posterior vaginal wall, uterus, cervix, or vaginal apex, into or beyond the vaginal canal due to weakening of the pelvic floor support. It can significantly impair quality of life and often requires surgical intervention for durable correction [1]. Sacrocolpopexy is considered the gold standard procedure for prolapse repair, traditionally performed via open, laparoscopic, or multi-port robotic approaches [2]. However, these techniques require transabdominal access and are associated with visible scars. Transvaginal natural orifice transluminal endoscopic surgery (vNOTES) is an emerging minimally invasive technique that allows access to the pelvic cavity through the vagina, thereby avoiding abdominal incisions, reducing postoperative pain, promoting rapid recovery, and achieving superior cosmetic outcomes [3].

Since its introduction, vNOTES sacrocolpopexy (vNOTES-SC) has evolved rapidly. Liu et al. first reported a pilot series of 26 cases of traditional laparoscopic vNOTES-SC, demonstrating significant improvement in anatomic support, as measured by the pelvic organ prolapse quantification (POP-Q) system, and in quality of life, as assessed by the Pelvic Floor Impact Questionnaire (PFIQ-7), with minimal morbidity [4]. Subsequently, standardized operative steps, including hydrodissection, Y-mesh tailoring, anterior mesh anchoring prior to prolapse reduction, and retroperitoneal tunneling, were described to enhance safety and reproducibility [5]. Comparative studies have shown that vNOTES sacrocolpopexy achieves outcomes comparable to laparoscopic approaches while offering shorter hospital stays and scarless recovery, with long-term data demonstrating low mesh exposure (0-6.5%), low recurrence rates (0-9%), high anatomical success (~90% beyond 24 months), and procedural proficiency achieved after approximately 11 cases based on cumulative sum analysis [6-11].

To address ergonomic and suturing challenges inherent in conventional vNOTES, robotic multiple-port-assisted vNOTES (RMP-vNOTES) was introduced. Guan et al. reported the first RMP-vNOTES (Xi platform) sacrocolpopexy, demonstrating improved dexterity and visualization during presacral dissection and mesh fixation [12,13]. However, multi-port robotic docking still resulted in external-arm collisions and spatial constraints within the transvaginal corridor.

The da Vinci single-port (SP) system integrates a flexible three-dimensional endoscope and three elbowed robotic instruments through a single multi-channel cannula, permitting consolidated docking via a 2.5-4 cm vaginal incision. The first robotic single-port vNOTES (RSP-vNOTES) hysterectomy in 2023 demonstrated feasibility and favorable perioperative outcomes [14]. However, the application of this platform in transvaginal sacrocolpopexy has not been well established.

## Case presentation

A 58-year-old Asian woman (gravida 3, para 2, abortus 1) presented with a one-year history of symptomatic uterine prolapse. She reported a sensation of vaginal bulge, pelvic pressure, and discomfort that progressively worsened over time and interfered with daily activities, including prolonged standing and physical exertion. She also described urinary and defecatory difficulty. Menopause occurred at age 53 years, and she denied postmenopausal bleeding.

**Figure 1:**
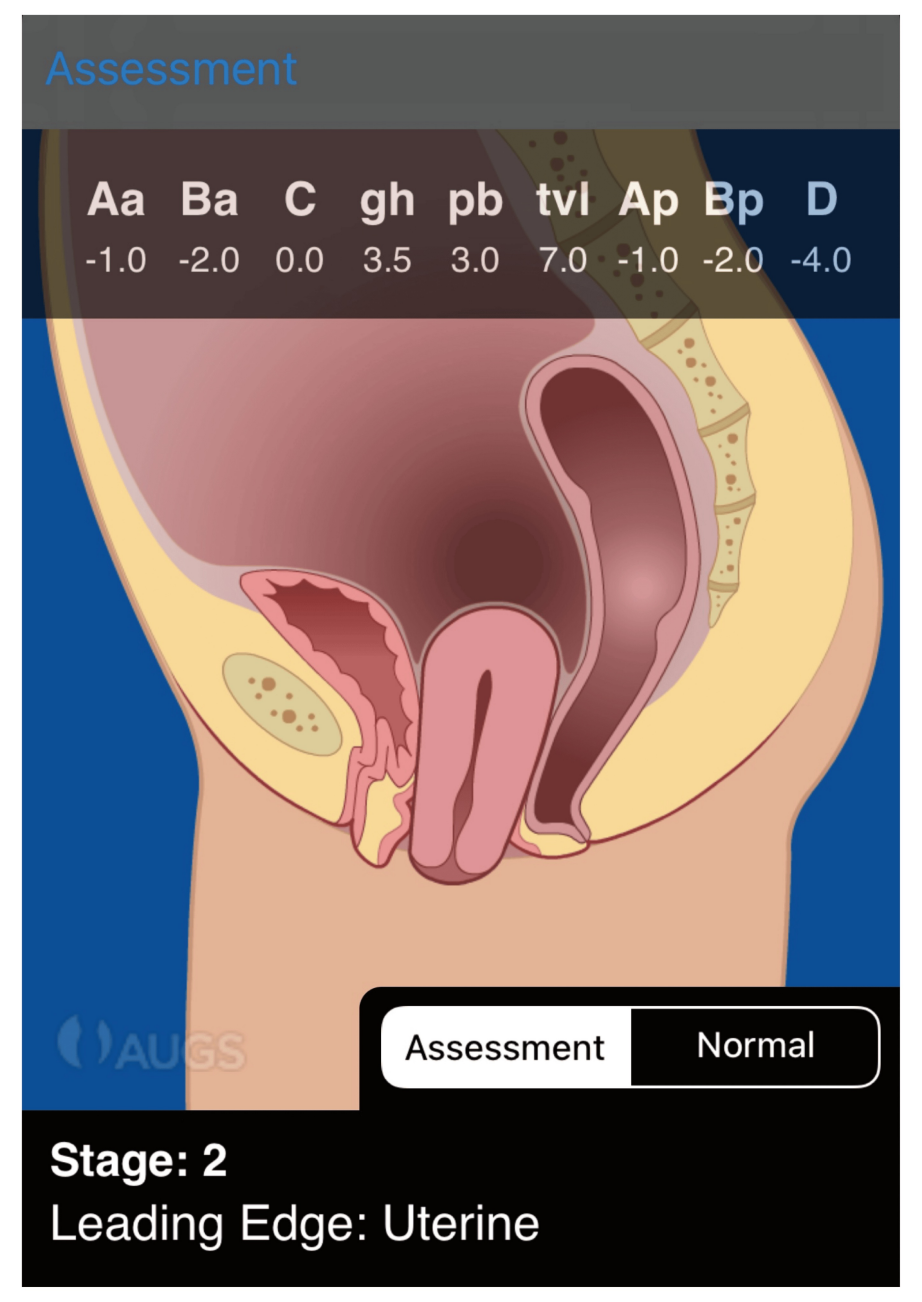
Preoperative pelvic organ prolapse quantification (POP-Q) assessment. Schematic representation of the preoperative pelvic organ prolapse quantification (POP-Q) examination demonstrating stage II uterine prolapse. Measured points are as follows: Aa -1.0 cm, Ba -2.0 cm, C 0.0 cm, Ap -1.0 cm, Bp -2.0 cm, and D -4.0 cm. Genital hiatus (gh) measured 3.5 cm, perineal body (pb) 3.0 cm, and total vaginal length (tvl) 7.0 cm. The leading edge of prolapse was the cervix at the level of the hymen, consistent with stage II apical prolapse. This image was created using the Pelvic Organ Prolapse Quantification (POP-Q) Interactive Assessment Tool, a registered trademark of Tim Peters and Company, Inc.

Pelvic examination revealed a well-developed vulva, smooth vaginal walls, and a cervix protruding beyond the introitus. The uterus was normal in size and mobile; the adnexa were soft and nontender. The pelvic organ prolapse quantification (POP-Q) assessment demonstrated stage II uterine prolapse, detailed in Figure 1. Preoperative evaluation was unremarkable. Cervical co-testing performed in June 2024 was negative for high-risk human papillomavirus.

Given the patient’s symptomatic burden and desire for definitive surgical management, treatment options, including conservative management, abdominal or transvaginal minimally invasive sacrocolpopexy, were discussed. After comprehensive counseling, the patient elected to undergo RSP-vNOTES hysterectomy with bilateral salpingo-oophorectomy and sacrocolpopexy using the da Vinci SP system, based on her preference for a minimally invasive, scarless approach and the surgical team’s expertise in robotic techniques.

**Figure 2:**
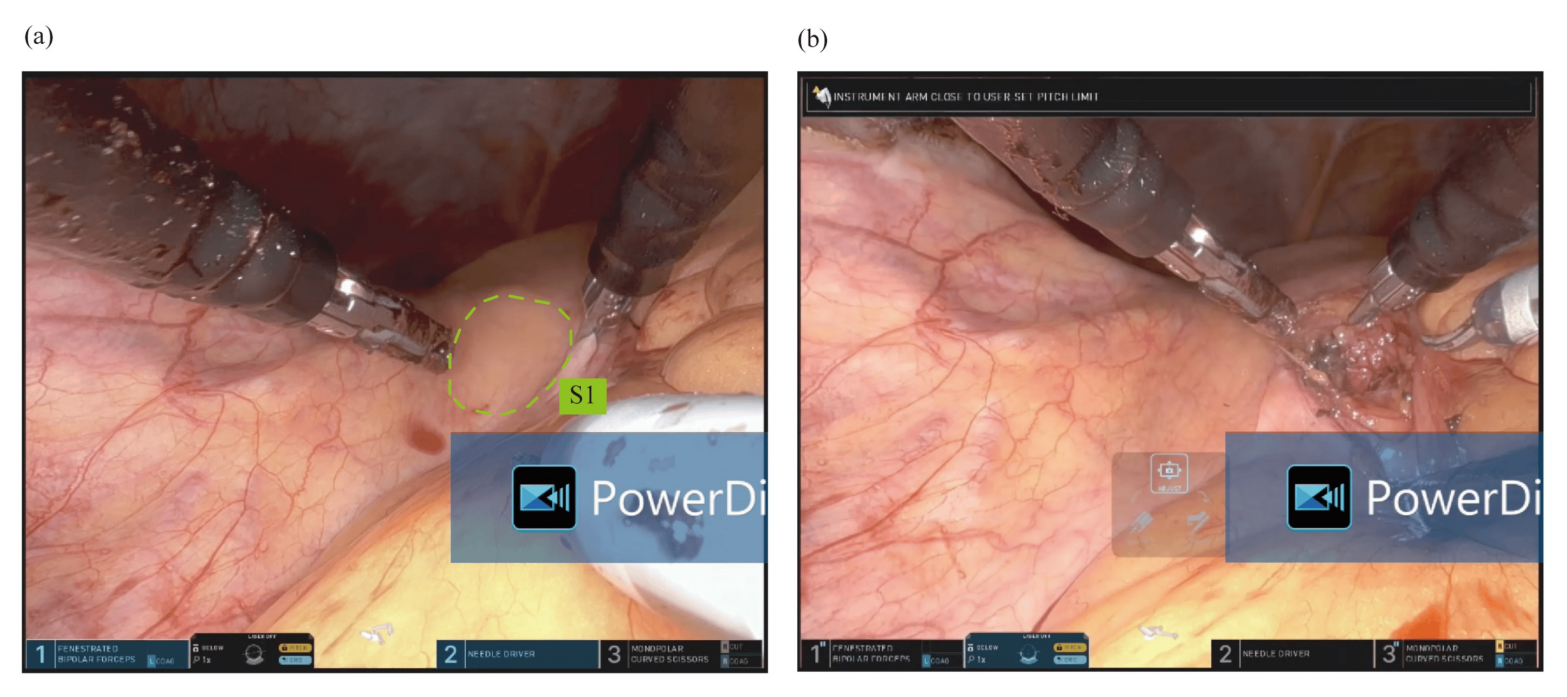
Identification of the sacral promontory. (a) The sacral promontory is visualized and anatomically localized. (b) The overlying peritoneum is incised to expose the anterior longitudinal ligament, a key fixation point for mesh placement.

**Figure 3:**
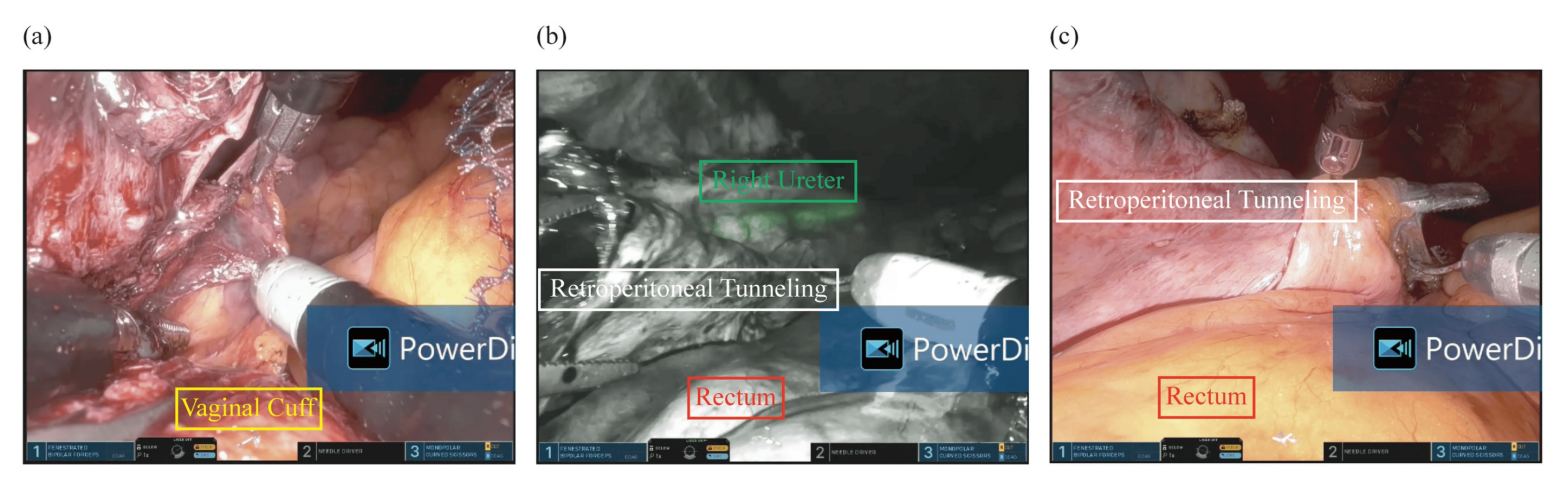
Retroperitoneal tunneling from the vaginal cuff to the sacral promontory. (a-b) A retroperitoneal tunnel (perirectal area) is created from the vaginal cuff toward the sacral promontory, facilitating mesh passage. (b) Indocyanine green (ICG) ureteral stent visualization under Firefly fluorescence mode to confirm ureteral location. (c) Completion of the retroperitoneal tunnel at the sacral promontory.

**Figure 4:**
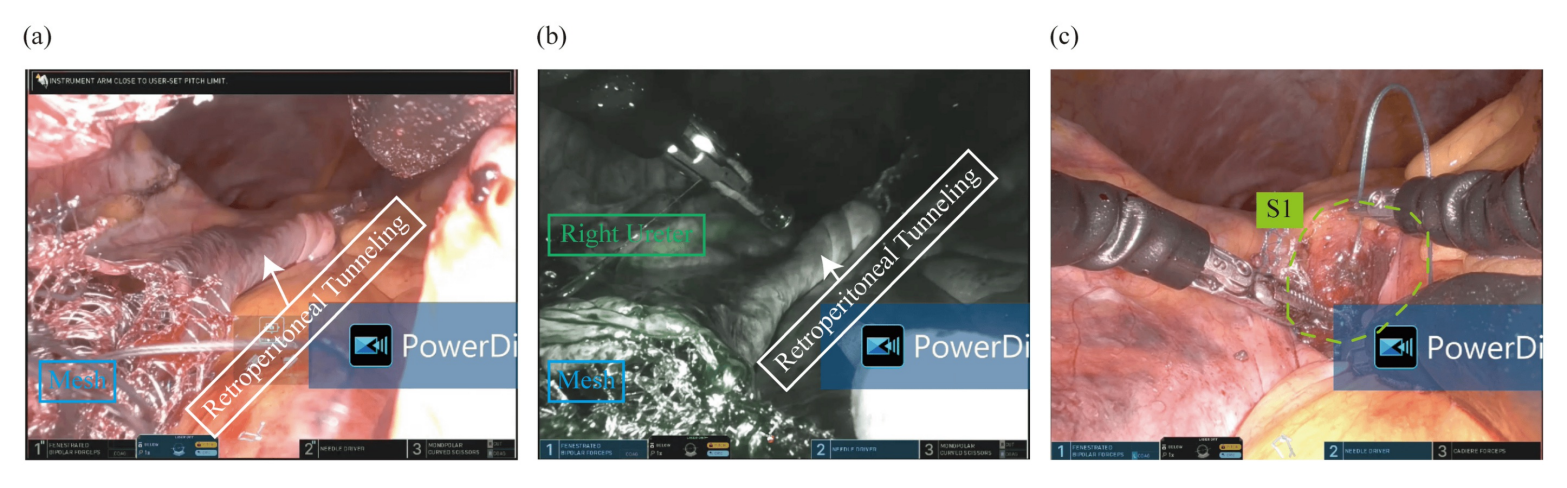
Y-mesh placement and sacral fixation. (a-b) The mesh is delivered through the retroperitoneal tunnel. (c) The proximal mesh arm is sutured to the anterior longitudinal ligament at the S1 level (green area).

Surgery was performed under general anesthesia on August 1, 2024. A single 2.5 cm transvaginal access port was placed, and the SP robotic cannula was docked. Following hysterectomy and adnexectomy, retroperitoneal tunneling to the sacral promontory was performed (Figures 2a, 2b, 3a-3c). A Y-shaped macroporous polypropylene mesh (Restorelle; Minneapolis, MN: Coloplast Corp.) was tailored intraoperatively based on anatomical measurements as follows: the anterior arm was trimmed to 5 cm according to the measured length of anterior vaginal dissection (plus 1 cm), and the posterior arm was trimmed to 14 cm based on the distance from S1 to the A point (16 cm), adjusted by subtracting 3 cm for posterior vaginal length and adding 1 cm to avoid excessive tension. Then, the tailored Y-shaped polypropylene mesh was secured to the anterior longitudinal ligament and affixed to both the anterior and posterior vaginal walls using 2-0 Ethibond sutures robotically (Figures 4a-4c). Peritoneal closure using 3-0 v-loc was completed robotically. After hemostasis was confirmed, the robot was undocked. Pneumoperitoneum was released with the AirSeal system turned off, and residual gas was evacuated. The access port was then removed from the vagina. The vaginal cuff angles were secured with figure-of-eight sutures using 0 Vicryl, and the cuff was closed circumferentially with 2-0 Vicryl to ensure hemostasis.

Total operative time was 170 min with estimated blood loss of 25 mL. No intraoperative complications occurred. The postoperative course was uneventful, and the patient was discharged on postoperative day two. At early postoperative follow-up and at one-year evaluation, the POP-Q assessment demonstrated stage 0 support with complete resolution of prolapse symptoms. No mesh exposure or recurrence was observed.

## Discussion

This case represents an early reported application demonstrating the technical feasibility and favorable short-term outcomes of robotic-assisted single-port vNOTES sacrocolpopexy for apical prolapse repair. The da Vinci SP platform integrates three-wristed robotic instruments and a flexible three-dimensional camera through a single transvaginal cannula, overcoming the spatial limitations inherent to conventional laparoscopic vNOTES and multi-port robotic approaches. In the present case, operative time (170 min) and estimated blood loss (25 mL) were comparable to those reported in previously published vNOTES sacrocolpopexy series, which have demonstrated median operative times of approximately 180-190 min and minimal blood loss [4,5,8]. Prior studies have also reported significant improvements in anatomical support and in quality of life, providing a standardized framework for outcome evaluation [4]. Although limited to a single case, our findings of favorable perioperative outcomes and sustained anatomical support at one-year follow-up are consistent with the existing literature.

Clinical evidence and technical evolution of vNOTES sacrocolpopexy

Since its initial description, vNOTES sacrocolpopexy (vNOTES-SC) has evolved from proof-of-concept feasibility to comparative effectiveness, durability assessment, and structured learning-curve analysis. The published clinical outcomes of vNOTES sacrocolpopexy are summarized in Table 1. The earliest pilot study of 26 patients demonstrated safe presacral dissection and mesh fixation to the anterior longitudinal ligament, confirming procedural feasibility and short-term anatomical correction [4]. Subsequent technical refinements, including hydrodissection, Y-mesh tailoring, anterior mesh anchoring before prolapse reduction, and retroperitoneal tunneling, standardized the operative approach and improved reproducibility [5]. Additional case series confirmed the feasibility of both traditional and robotic multiple-port-assisted vNOTES (RMP-vNOTES) sacrocolpopexy, with acceptable operative times and low perioperative morbidity [8,13].

**Table 1:** Summary of published studies on vNOTES sacrocolpopexy. NA: not available; vNOTES: transvaginal natural orifice transluminal endoscopic surgery; LESS: laparoendoscopic single-site surgery; TS-LSC: transvaginal single-port laparoscopic sacrocolpopexy; LSC: laparoscopic sacrocolpopexy; TV-NOTES: traditional transvaginal natural orifice transluminal endoscopic surgery; RV-NOTES: robotic transvaginal natural orifice transluminal endoscopic surgery; CUSUM: cumulative sum

Studies	Design	Number of cases	Follow-up	Mesh exposure	Recurrence	Learning curve
Liu et al. (2019) [4]	Pilot case series	26	1 month	0%	0%	Standardized after case 4
Liu et al. (2019) [5]	Technical report	1	NA	NA	NA	Technique refinement
Chen et al. (2023) [6]	Propensity-matched (vNOTES vs. LESS)	36 vNOTES	48.6 months	2.8%	0%	NA
Li et al. (2022) [7]	Retrospective cohort (TS-LSC vs. LSC)	46 TS-LSC	~39 months	6.5%	2.2% (no apical)	NA
Mei et al. (2023) [8]	Case series	8 TV-NOTES and 2 RV-NOTES	Up to 2 years	0%	0%	Feasible for beginners
Lu et al. (2022) [9]	Retrospective cohort	55	35.5 months	5.5%	5.5%	NA
Ilter et al. (2025) [10]	Multi-center retrospective	23 vNOTES	21 months	NA	8.7%	NA
Liu et al. (2025) [11]	Cohort+CUSUM	79	Median 73 months	3.8%	9% (anatomical cure 91%)	Inflection at case 11
Bulutlar et al. (2025) [15]	Prospective case series	8	90 days	0%	0%	Short operation time (62 min)
Erkmen and Arkan (2025) [16]	Retrospective cohort	30	12 months	NA	6.7%	NA

Comparative studies further expanded the evidence base. A propensity score-matched analysis demonstrated that vNOTES-SC achieved similar mid-term outcomes compared with laparoendoscopic single-site surgery (LESS) sacrocolpopexy while reducing operative time and hospital stay [6]. Transvaginal single-port laparoscopic sacrocolpopexy likewise showed non-inferiority to conventional laparoscopy with improved cosmetic satisfaction and lower postoperative pain scores [7]. Long-term follow-up data demonstrated mesh exposure rates ranging from 0% to 6.5% and recurrence rates from 0% to 9%, with most studies reporting anatomical success approaching 90% beyond 24 months [8,9]. Multi-center evaluations confirmed favorable mid-term outcomes compared with native tissue suspension techniques, emphasizing appropriate patient selection [10]. Learning-curve analysis using the cumulative sum (CUSUM) methodology demonstrated procedural proficiency after approximately 11 cases, with stable operative time and complication rates thereafter [11]. Contemporary technique modifications, such as the Kılıççı-Bulutlar approach, have further optimized operative efficiency [15]. Additionally, recent cohorts evaluating concomitant hysterectomy with vNOTES-assisted sacrocolpopexy have reinforced the safety and feasibility of this scarless approach [16].

Collectively, more than 300 patients have undergone vNOTES sacrocolpopexy across published studies, demonstrating consistent perioperative outcomes, short hospitalization (one to two days), and durable apical support. These data position vNOTES-SC as a reproducible, minimally invasive alternative to conventional laparoscopic sacrocolpopexy.

The integration of robotic technology marked the next phase in this evolution. Robotic vNOTES sacrocolpopexy using the da Vinci Xi platform was first reported in 2020-2021, demonstrating that wristed instrumentation and three-dimensional visualization improved suturing precision at the anterior longitudinal ligament and enhanced ergonomics during sacral promontory dissection [12,13]. Early robotic-assisted vNOTES cases confirmed safe transvaginal docking and successful anatomic restoration [13]. Subsequent comparisons between traditional and robotic-assisted vNOTES showed comparable safety while acknowledging longer operative times during early adoption [8]. These experiences established the technical groundwork for the transition toward a consolidated single-port robotic platform.

Robotic single-port (SP) vNOTES surgery - clinical evolution and publications

RSP-vNOTES represents the latest technological advancement in the evolution of minimally invasive vaginal surgery. Following the establishment of conventional laparoscopic vNOTES and subsequent integration of robotic multi-port (Xi) platforms, the da Vinci SP system was introduced to address ergonomic limitations associated with parallel multi-arm docking within the confined transvaginal corridor. These challenges include external arm collision, restricted triangulation, and limited intracorporeal maneuverability.

The first reported RSP-vNOTES hysterectomy using the da Vinci SP platform was described in 2023 as a standardized 10-step procedure, demonstrating technical feasibility, safe docking, and same-day discharge without perioperative complications [14]. Shortly thereafter, a 28-patient feasibility series confirmed reproducibility, with acceptable operative times, minimal blood loss (mean 32 mL), and low complication rates, supporting early clinical adoption [17].

International expansion followed in 2024 with the first reported use of a domestically developed Chinese robotic SP system for transvaginal hysterectomy, demonstrating feasibility, favorable postoperative recovery, and confirming platform generalizability beyond the Intuitive system [18]. Comparative effectiveness evidence emerged in 2025. A large retrospective cohort study of 383 patients comparing RSP-vNOTES and RMP-vNOTES hysterectomy demonstrated no significant differences in operative time, blood loss, or complication rates after adjustment, while highlighting ergonomic advantages of the SP platform, particularly in complex endometriosis cases [19]. Similarly, a propensity score-matched comparison between RSP-vNOTES and conventional laparoscopic vNOTES hysterectomy demonstrated equivalent perioperative outcomes [20].

Expanded indications were demonstrated in a video report of vaginal-assisted NOTES hysterectomy for a 970 g uterus using the SP system, confirming technical feasibility in anatomically challenging cases and emphasizing improved dexterity and visualization associated with the single-cannula design [21]. Objective learning-curve analysis of 97 consecutive RSP-vNOTES hysterectomies showed stabilization of operative time after approximately 30 cases using cumulative sum (CUSUM) methodology, with low conversion and complication rates throughout the adoption phase [22]. More recently, the application of RSP-vNOTES has extended into gynecologic oncology, with a successful robotic single-port omentectomy performed for ovarian granulosa cell tumor staging, demonstrating the feasibility of upper abdominal dissection through a transvaginal approach [23].

A summary of the current literature on RSP-vNOTES is presented in Table 2. Collectively, these publications illustrate a structured developmental trajectory as follows: (1) initial feasibility, (2) early validation and international adoption, (3) comparative effectiveness research, (4) expansion to complex and large uterine pathology, (5) learning-curve characterization, and (6) oncologic application.

**Table 2:** Summary of published studies on RSP-vNOTES. Short-term follow-up was defined as <6 months, mid-term as six to 12 months, and long-term as ≥12 months. RSP-vNOTES: robotic single-port transvaginal natural orifice transluminal endoscopic surgery; RMP-vNOTES: robotic multiple-port transvaginal natural orifice transluminal endoscopic surgery; T-vNOTES: traditional transvaginal natural orifice transluminal endoscopic surgery; SP: single-port

Studies	Study design	Sample size (n)	Follow-up	Key findings
Guan et al. (2024) [14]	Pioneering case	1	Short-term	First RSP-vNOTES hysterectomy
Guan et al. (2024) [17]	Case series	28	Short-term	Feasible, blood loss 32 mL
Zhang et al. (2024) [18]	Case report (Chinese SP platform)	2	Short-term	Platform generalizability
Yang et al. (2025) [19]	Retrospective cohort (RSP-vNOTES vs. RMP-vNOTES)	383 (92 RSP-vNOTES)	Short-term	Comparable to RMP-vNOTES
Kanno et al. (2025) [20]	Propensity-matched (RSP-vNOTES vs. T-vNOTES)	184 (65 RSP-vNOTES after PSM)	Short-term	Equivalent to T-vNOTES
Kanno et al. (2025) [21]	Video article (large uterus)	1	Short-term	970 g uterus feasible
Yang et al. (2026) [22]	Learning-curve study	97	Short-term	Proficiency achieved plateau ~30 cases
Zhang et al. (2025) [23]	Oncologic case	1	Short-term	Omentectomy feasible

Technically, the SP system delivers three fully wristed robotic instruments and a flexible articulating three-dimensional camera through a single multi-channel cannula. This configuration restores true intracorporeal triangulation within the vaginal corridor, minimizes external arm collision, reduces instrument crowding, and enhances exposure in deep pelvic spaces. For reconstructive procedures such as sacrocolpopexy, the SP platform facilitates controlled retroperitoneal tunneling and precise mesh fixation to the anterior longitudinal ligament under stable magnified visualization.

From a patient-centered perspective, RSP-vNOTES preserves the core advantages of natural orifice surgery, including the absence of abdominal incisions, reduced postoperative pain, early ambulation, and rapid recovery. From a surgeon's perspective, improved ergonomics and centralized docking may reduce fatigue and promote consistent instrument control during complex pelvic reconstruction.

Despite encouraging perioperative and early adoption data, the current literature remains predominantly retrospective and single-center. Long-term durability data for reconstructive procedures such as sacrocolpopexy are limited. Prospective multi-center studies, standardized outcome definitions, and cost-effectiveness analyses are required to more definitively establish the role of RSP-vNOTES within the minimally invasive surgical paradigm.

The present case extends the evolution of vNOTES sacrocolpopexy into the era of robotic single-port surgery and demonstrates that complex apical suspension can be safely achieved through a completely scarless transvaginal approach using advanced robotic articulation.

## Conclusions

RSP-vNOTES sacrocolpopexy appears to be a safe, feasible, and cosmetically advantageous option for apical prolapse repair. In this case, anatomical and functional outcomes remained optimal at one-year follow-up. The da Vinci SP platform enhances exposure, retroperitoneal tunneling, and suturing through its tri-arm configuration and stable three-dimensional optics. Continued clinical experience and prospective studies are warranted to confirm long-term durability and to further define the role of RSP-vNOTES in the future of minimally invasive pelvic reconstructive surgery.
